# Exposure to Ambient NO_2_ Increases the Risk of Dry Eye Syndrome in Females: An 11-Year Population-Based Study

**DOI:** 10.3390/ijerph18136860

**Published:** 2021-06-26

**Authors:** Chi-Jung Chung, Ning-Yi Hsia, Chih-Da Wu, Ting-Ju Lai, Jein-Wen Chen, Hui-Tsung Hsu

**Affiliations:** 1 Department of Public Health, China Medical University, Taichung 406040, Taiwan; cjchung1010@gmail.com (C.-J.C.); routinelai@gmail.com (T.-J.L.); 2 Department of Medical Research, China Medical University Hospital, Taichung 406333, Taiwan; 3 Department of Ophthalmology, China Medical University Hospital, Taichung 406333, Taiwan; deepwhite1111@hotmail.com; 4 Department of Geomatics, National Cheng Kung University, Tainan 701001, Taiwan; chidawu@mail.ncku.edu.tw; 5 National Institute of Environmental Health Sciences, National Health Research Institutes, Miaoli 350401, Taiwan; 6 Department of Food and Beverage Management, Cheng Shiu University, Kaohsiung 833301, Taiwan; jwchen@gcloud.csu.edu.tw; 7 Center for Environmental Toxin and Emerging-Contaminant Research, Cheng Shiu University, Kaohsiung 833301, Taiwan; 8 Super Micro Mass Research and Technology Center, Cheng Shiu University, Kaohsiung 833301, Taiwan

**Keywords:** air pollution, PM_2.5_, NO_2_, dry eye syndrome, land-use regression model

## Abstract

Previous studies have indicated that women suffer from dry eye syndrome (DES) more significantly than men. Therefore, we specifically explore the associations between air pollutant levels and the risks of DES for women. The study obtained 27,605 participants from the 29 recruitment centers of the Taiwan Biobank, which was established in October 2012. A large scale cross-sectional study involving DES sufferers and age- and education-matched control groups without DES was designed. Based on the municipality of residence, the predicted concentration levels of various air pollutants, including PM_2.5_, sulfur dioxide (SO_2_), ozone (O_3_), and nitrogen dioxide (NO_2_) were estimated by using hybrid kriging/LUR model. Multiple logistic regressions were applied to estimate the prevalence ratios (PR) of DES and 95% confidence interval. Hormone supplementations, DBP, allergies, and arthritis were considered as important comorbidities for increased PR risk of DES. In addition, with each standard deviation (SD) increment of PM_2.5_ and temperature, women had significant increases in PRs of DES of 1.09- and 1.07-fold, respectively; conversely, each SD increment of relative humidity (RH) had a protective effect against the risk of DES. After considering hormone supplementation, arthritis, and allergy, the SD increment of NO_2_ and temperature were associated with the PRs of DES. In conclusion, significant associations of ambient NO_2_ concentration, RH and temperature with DES indicated the importance of increased environmental protection in the female population. Female exposure to high levels of NO_2_ when receiving hormone supplementation, or suffering with allergies or arthritis, had significantly increased risk of DES.

## 1. Introduction

Dry eye syndrome (DES) is a common, multi-factorial, ocular surface disorder of increasing prevalence that involves symptoms of ocular discomfort, fluctuating visual acuity, and tear film instability with the potential to damage the ocular surface [1]. Because it can affect daily activities and also causes deterioration in quality of life, it is an important public health issue. It is estimated that about 7.8% (3.23 million) of American women and 4.7% (1.68 million) of American men aged 50 and older are affected by DES [2,3]. The prevalence of DES in some Asian countries is particularly high, ranging from 4.87% to 61.57% [4,5,6,7,8,9]. According to different survey methods, the recorded prevalence of DES in Taiwan ranges from 4.87% to 34% [8,9].

Several common risk factors have been repeatedly identified as being associated with DES. These include age, gender, cigarette smoking, and low body mass index [10,11,12]. In addition, hypertension, serum total to high-density lipoprotein (HDL) cholesterol ratio, diabetes, history of arthritis, osteoporosis, gout, thyroid disorder, presence of any allergy, asthma, and stroke are also considered to be related to the prevalence of DES [11,13,14]. Among the risk factors for DES, women have repeatedly been found to be at increased risk of DES compared with men [10,12,13,14,15]. A Taiwanese nationwide dataset (2001–2015) also showed that females had a significantly higher annual incidence rate of DES than males [16]. Furthermore, previous studies have indicated that postmenopausal women and women receiving hormone therapy have more severe cases of dry eye [13,15,17,18].

Studies have also indicated that air pollution is associated with DES [19,20,21]. Owing to the structure of the ocular surface, only a very thin tear film separates the corneal and conjunctival epithelia from air pollutants. Therefore, it is reasonable to assume that the ocular surface system can be affected by air pollution, leading to a large variety of clinical signs and symptoms [22].

Unfortunately, studies on the relationship between air pollution and DES have presented inconsistent results. For example, Mo et al. (2018) conducted a case-crossover study to investigate the impacts of air pollution on DES among residents in Hangzhou, China. Their results showed that air pollutants (PM_10_, PM_2.5_, SO_2_, NO_2_, and CO) were significantly associated with DES outpatient visits [20]. However, research in Taiwan showed that DES is only related to the concentrations of CO and NO_2_ [21], while research in South Korea showed that DES is associated with the concentrations of O_3_ and NO_2_ [19]. None of these studies considered whether female subjects were receiving hormone therapy or adjusted for this possibility in the analysis. It is thus considered necessary to undertake a more detailed analysis of this factor in order to identify the impact of air pollution on DES.

Another important issue when studying the impact of environmental factors on DES is that location-based air pollution data frequently have a far lower resolution than location-based health data. When integrating these two types of data to characterize the relationship between exposure and health, there will be variation in the spatial correspondence between the two [23]. In this study, we used a previously developed hybrid kriging/land use regression (LUR) model to improve the accuracy in predicting the concentrations of air pollution and to enhance the quality of characterization of exposure [24]. Therefore, the purpose of this study is to analyze the risk factors of DES in the population most sensitive to it, namely women, and to investigate whether a higher level of ambient air pollution is associated with an increased risk of DES.

## 2. Materials and Methods

The data were obtained from the Taiwan Biobank, which was established in October 2012 with the aim of identifying the potential risk factors of various diseases through combining information of genome-wide analysis, methylation arrays, lifestyle, and clinical biochemistry (https://www.twbiobank.org.tw/new_web/about.php, accessed on 17 May 2019). A total of 29 recruitment centers were established in Taiwan and the process of recruitment was performed in accordance with the relevant guidelines and regulations. This study was approved by the Research Ethics Committee of China Medical University Hospital, Taichung, Taiwan (CRREC-108-006), and adhered to the principles of the Declaration of Helsinki. Written informed consent was obtained from all of the participants prior to data collection. Academics can apply to obtain the dataset of the Taiwan Biobank.

### 2.1. Study Participants

**Figure 1:**
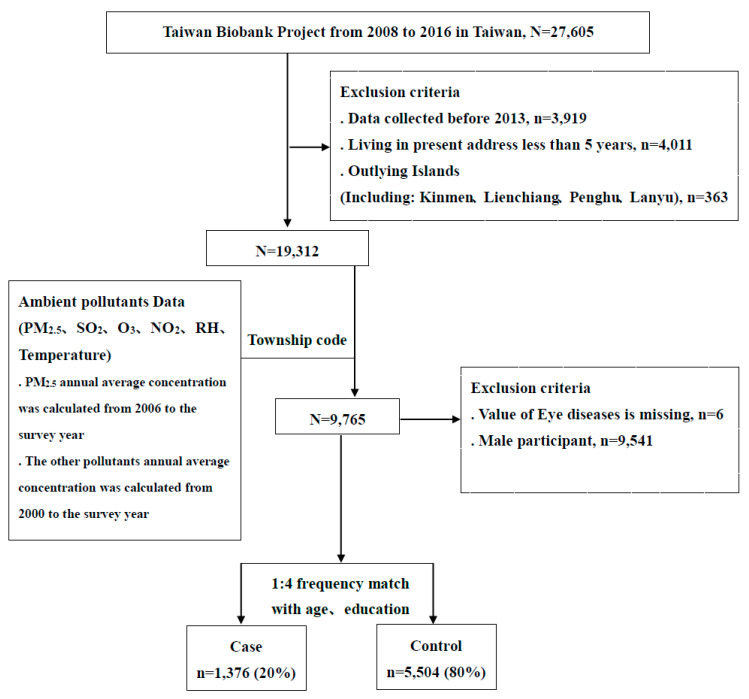
Detailed flow chart of the recruitment procedure in this study.

The original dataset included 27,605 participants aged 30–70 years old with no history of cancers. After excluding cases with incomplete data before the recruitment year 2013 (*n* = 3919), people living at their present address for less than five years (*n* = 4011), people living on outlying islands (*n* = 363), males (*n* = 9541), as well as those with missing information regarding eye diseases (*n* = 6), there were 9765 participants for inclusion in this study. The study participants were defined as having DES if they answered “Yes” to the question, “Have you ever had DES?” in the questionnaire of individual disease history. We further adopted a ratio of 1:4 for cases and controls without DES, matching for age and education. There were 1376 cases with DES and 5508 controls in the final analysis. A detailed flow chart of the recruitment procedure is shown in Figure 1.

### 2.2. A Hybrid Kriging/Land-Use Regression (LUR) Model for Ambient Air Pollutants Estimation

We adopted the air pollutant data collected from air quality monitoring stations in Taiwan between 2006–2018, including PM_2.5_, sulfur dioxide (SO_2_), ozone (O_3_), and nitrogen dioxide (NO_2_), as well as relative humidity and temperature, to calibrate a hybrid kriging/LUR model. We calculated the predicted concentration level from kriging interpolation as a variable in the LUR model to improve the accuracy of predicting the variation of various air pollutants. The spatial–temporal resolution of the modeled air pollution in this study was a 50 m × 50 m grid size with daily averaged pollutant level. We then aggregated to annual average for assessing its long-term effects on DES. The cross-validated R^2^ values were 0.61 for PM_2.5_, 0.63 for NO_2_, 0.20 for O_3_, and 0.61 for SO_2_. The details of the procedure and model validation are published in previous papers [25,26]. ArcView GIS (version 93, ESRI Inc., Redlands, CA, USA) and its Geostatistical Analysts Extension (ESRI Inc., Redlands, CA, USA) were used in the constructed model. To protect the privacy of the subjects in terms of their addresses, the resultant LUR model was applied to estimate the levels of air pollutants for every resident based on the municipality of residence. Finally, we calculated the overall mean values of PM_2.5_, SO_2_, NO_2_, O_3_, relative humidity, and temperature for every resident from the earliest acquired year for air pollution, 2006 for PM_2.5_ and 2000 for other pollutants, to their corresponding year of recruitment (2013–2017).

### 2.3. Collection of Questionnaires and Health Examinations

All study participants were invited to undergo baseline heath examinations including height, weight, waist circumference, pulse, and vision. In addition, systolic blood pressure (SBP), diastolic blood pressure (DBP), blood glucose, HbA1c%, and blood lipid profiles such as total cholesterol (TC), triglyceride (TG), high-density lipoprotein (HDL) cholesterol, low-density lipoprotein (LDL) cholesterol, and estimated glomerular filtration rate (eGFR) were measured and acquired from the database. The questionnaires included questions on demographic characteristics and lifestyle factors, such as cigarette smoking (yes/no), drinking alcohol (yes/no), tea (yes/no), or coffee (yes/no), and performing regular exercise (yes/no), among others. Data on history of pregnancy, hormone supplementation (yes/no), and menopause (yes/no) were also collected for females. In addition, individual and family histories of disease were acquired through face-to-face interviews.

### 2.4. Statistical Analysis

Data are presented as number (percentage) and mean (±standard deviation) for categorical and continuous variables, respectively. Receiver operating characteristic (ROC) curve analysis was performed to define the optimal cut-off points for individual indices of air pollutants between those with and without DES. The cut-off points of all indices exhibited the largest values of the area under the curve (AUC) and Youden index. The relevant risk factors are presented in Table 1 and Table 2 and were used in the stepwise logistic regression to identify the relevant confounders, including age, education, hormone supplementation, arthritis, and allergy. In addition, multiple logistic regression analysis was used to evaluate the prevalence ratios (PRs) of DES as well as the interaction of all indices of air pollutants and disease history (hormone supplementation, arthritis, and allergy) on the PRs of DES after adjusting for relevant confounders. Finally, we also used stepwise logistic regression analysis to identify the factors important for an increased risk of DES.

## 3. Results

The average ages of participants with DES and controls were 53.89 and 53.55 years, respectively (Table 1). More than 60% of the study participants were older than 50 and approximately 90% of the participants had an education level of high school, college, or above. Subjects living in central Taiwan had a significantly increased PR of DES (1.67-fold; 95% CI: 1.21–2.32). Similarly, subjects living in southern Taiwan also had a significantly increased PR of DES (1.46-fold; 95% CI: 1.05–2.02). Women showed less of a tendency to smoke and drink alcohol, but more of a tendency to drink tea or coffee and play sports. However, there were no associations between these habits and the PR of DES. Irrespective of the presence or absence of DES, 60% of females had undergone menopause and about 20% had taken hormone supplements, including treatments of menopause, certain diseases, or as contraception. Our data showed that undergoing hormone treatments in women had a significant PR of DES (1.55-fold; 95% CI: 1.22–1.95).

We explored the associations of the biochemical profile and individual disease history with DES, as shown in Table 2. Females with DES had lower levels of SBP and DBP than the controls; meanwhile, in subjects with high SBP, DBP, and BMI, as well as high waist-to-hip ratio (per unit increment), there were significant protective effects against the risk of DES, with its PR ranging from 0.86 to 0.93. However, we did not observe any associations of blood glucose, lipid profile, eGFR, or lung function with the PR of DES. Regarding the individual disease of history, we found that females with an allergy, osteoporosis, and arthritis had significantly increased risk of DES (PR = 1.30, 95% CI: 1.09–1.55 for allergy, PR = 1.46, 95% CI: 1.19–1.80 for osteoporosis and PR = 2.00, 95% CI: 1.64–2.43 for arthritis). However, there were no associations between gout or asthma and risk of DES in the present analysis.

Females with DES had significantly high levels of exposure to PM_2.5_ and NO_2_, and conditions with high temperature and low relative humidity (all *p* values < 0.05 through Wilcoxon rank-sum tests, data not shown) (Table 3). After adjusting for age and education, each standard deviation (SD) increment of PM_2.5_, SO_2_, NO_2_, and temperature significantly increased the PR of DES by 1.09-, 1.05-, 1.06-, and 1.07-fold, respectively. We further considered all relevant risk factors in Table 1 and Table 2 in stepwise logistic regression analysis, and found that the most important factors were hormone supplementation, arthritis, and allergy. Therefore, we adjusted for these factors in the subsequent multiple logistic regression analysis. After adjusting for age, education, hormone supplementation, arthritis, and allergy, there were no associations between these indices and the risk of DES. Because PM_2.5_ was the only pollutant with an annual average value greater than the recommended values of the Taiwan Environmental Protection Agency (annual average of 15 g/m^3^), we used the ROC curve estimation method to determine the appropriate air pollutant cut-off points. The cut-off points of PM_2.5_, SO_2_, NO_2_, O_3_, relative humidity, and temperature were 28.13 (AUC: 0.5245, *p* = 0.0043), 4.61 (AUC: 0.5052, *p* = 0.5570), 17.02 (AUC: 0.5171, *p* = 0.0462), 28.89 (AUC: 0.5085, *p* = 0.3280), 78.08 (AUC: 0.5275, *p* = 0.0016), and 21.81 (AUC: 0.5224, *p* = 0.0090), respectively. We then defined the high-exposure group as individuals with levels of exposure to air pollutants at concentrations higher than their individual cut-off points. We found that individuals with high exposure to air pollutants, including PM_2.5_, SO_2_, NO_2_, and O_3_, as well as high temperature, had significantly increased PRs of DES of 1.15–1.40-fold after adjusting for age and education. In addition, exposure to a high level of relative humidity was associated with a protective effect against the risk of dry eye syndrome. Finally, after considering other confounding factors, two parameters were found to be associated with increased PR of DES: NO_2_ (PR = 1.43; 95% CI: 1.15–1.78) and temperature (PR = 1.44; 95% CI: 1.09–1.89).

In addition, we executed a sensitivity analysis to evaluate the effects from different exposure duration of 1 year, 3 years, and 5 years before the survey day on the PR of DES in Supplementary Table S2. The results showed as similar to those in Table 3, in which NO_2_ was still the most predominated air pollutant affecting the PR of DES after considering other confounding factors.

We further evaluated the interactions of various air pollutants, hormone supplementation, arthritis, as well as allergy on the PR of DES, as shown in Table 4. The results indicated that, in those exposed to high levels of PM_2.5_, SO_2_, NO_2_, and temperature, and with histories of hormone supplementation, arthritis, as well as allergy, there were significant dose-response relationships for increased PRs of DES. However, almost no positive interactions were observed in the present analysis. We only observed a borderline significant positive interaction between the levels of NO_2_ and arthritis (*p* = 0.0698). Individuals with high exposure to NO_2_ and arthritis had an increased PR of DES of 2.80-fold (95% CI: 1.88–4.16, *p* < 0.01).

We incorporated all relevant risk factors into a stepwise logistic regression model and attempted to find the most important factors increasing the risk of DES (Table 5). The results identified five factors, namely, hormone supplementation (PR = 1.47, 95% CI: 1.17–1.86), DBP per increment of SD (PR = 0.99, 95% CI: 0.98–1.00), allergy (PR = 1.36, 95% CI: 1.01–1.82), arthritis (PR = 1.76, 95% CI: 1.29–2.41), and high NO_2_ exposure (PR = 1.43, 95% CI: 1.15–1.78).

## 4. Discussion

Previous studies have indicated that women suffer from DES significantly more than men [10,12,13,14,15], and we obtained similar results in this study using the Taiwan Biobank dataset. We present the results of such analysis in Table S1, showing that women suffer from DES at a rate 2.98 times higher than that of men. This difference is statistically significant. This is supported by another national study in which the prevalence of dry eye in Taiwan was again shown to be higher in women than in men [16]. Because women have a higher prevalence of dry eye, we focused this study on women who are more sensitive to DES. In particular, recently, many studies have shown that women receiving hormone therapy are at risk for dry eye. We noticed that, in most studies discussing the relationship between air pollution and dry eye, there was no adjustment for the influence of this variable. Therefore, we specifically explored the association between air pollution and dry eye in women after adjusting for dry eye confounding factors. To the best of our knowledge, this study is the first to specifically explore the independent or dependent effects of the levels of various air pollutants on PRs of DES for women. In addition, to obtain high-resolution data on the exposure to different concentrations of air pollution, we used a hybrid kriging/LUR model to simulate the spatial distribution of such concentrations, as well as temperature and humidity, in this study. We also used the ROC curves to define the cut-off points between high and low exposure to the environmental parameters. In this study, we found that five factors had significant correlations with DES in females: receiving hormone therapy, diastolic blood pressure, allergies, arthritis, and exposure to NO_2_.

It has been demonstrated that, within human ocular tissues, androgen, estrogen, and/or progesterone receptor mRNAs are present in the lacrimal gland, meibomian gland, lacrimal gland acinar epithelial cells, palpebral and bulbar conjunctivae, and cornea. It is suggested that sex steroid receptor mRNA expression in ocular tissues may increase or decrease in response to changes in the sex hormone environment, such as those during aging [27]. It has also been found that high estrogen levels are associated with reduced tear function in postmenopausal women [28]. In addition, it has been suggested that estrogens exert an adverse pro-inflammatory effect on the ocular surface [18]. Currently, there is also a general consensus that low levels of circulating androgens and high levels of circulating estrogens are risk factors for DES [18]. In a large cohort study assessing the relationship between hormone replacement therapy (HRT) and DES, it was shown that the multivariable-adjusted odds ratios for DES were 1.69 (95% CI: 1.49–1.91) for estrogen use alone and 1.29 (95% CI: 1.13–1.48) for estrogen plus progesterone/progestin use, compared with no HRT. It was also found that the risk increased with a longer duration of HRT [29]. This was confirmed in a study conducted by Erdem et al. (2007), who also found that the use of HRT can increase the incidence of DES in postmenopausal women. In our study, comparing with women without hormone supplementation, women with such supplementation appeared to be at 47% increased risk of DES. Nonetheless, it is reported that there is still scientific controversy about the impact of hormone therapy on dry eye [17,18]. However, using systematic review and meta-analysis, Dang et al. (2019) concluded that a non-significant improvement in postoperative tear production, as well as tear breakup time, was seen after HRT treatment at follow-ups in dry eye patients [30]. The results of our work also suggested that it is necessary to consider the risk of DES for postmenopausal women who are receiving hormone medication.

In this study, the PR of DES showed a significant difference depending on the geographical area (Table 1). A preliminary comparison found that there was a relatively consistent trend in the association of DES with air pollution index (AQI). According to 2013–2018 data released by the Taiwan Environmental Protection Agency, the average AQI values of the North, Central, South, and East regions are 61.2 ± 6.2, 77.5 ± 9.0, 79.1 ± 6.4, and 41.4 ± 5.5, respectively. This suggested that air pollution plays a certain role in the PR of DES. In further analysis, we performed adjustments for all significant confounding factors including age, educational level, hormonal therapy, arthritis, and allergies; we found that the concentration of NO_2_ was significantly associated with the PR of DES (Table 4 and Table 5). Another study conducted in Taiwan also indicated that CO, NO_2_, and temperature were positively associated with DES [21]. The effect of chronic exposure to NO_2_ on the ocular surface was also explored by Novaes et al. (2010). Their results demonstrated that NO_2_ exposure is positively associated with the Ocular Symptom Disease Index score and reported ocular irritation. Furthermore, NO_2_ exposure was described as being negatively associated with tear breakup time (BUT) [31]. In a case-control study, researchers found that a group with high exposure to traffic pollution had significantly less tears travelling on filter paper (Schirmer’s test) than a control group. Moreover, the BUT for the high-exposure group was also significantly lower than that of the control group [32].

It is suggested that the core mechanism of DES is inflammation [33]. Nitrogen oxides are irritant gases with low water solubility. McKay’s study demonstrated that lung tissue could be damaged by nitrogen oxides due to the generation of reactive nitrogen-derived free radicals. Furthermore, nitrogen oxides produce nitric acid after contact with water and continue to stimulate the inflammation of lung tissue [34]. In an elderly cohort study, it was also found that an interquartile range increase in 24 h exposure to NO_2_ was associated with a 1.7% (95% CI, 0.2%–3.3%) increase in fibrinogen [35]. Fibrinogen is a key regulator of inflammation in disease [36]. It is considered that the pathophysiology is the same in the eye as that in the lung, where conjunctiva as a mucous membrane that covers the sphere is irritated by reactive nitrogen-derived free radicals and nitric acid. This causes chronic inflammation of the eye and plays a key role in DES. The results of this study showed that DES is a subclinical form of ocular inflammation that occurs as a consequence of exposure to NO_2_.

In the multiple logistic regression model, subjects exposed to a high temperature had a significantly higher PR of DES than the low temperature exposure group (Table 4). Another Taiwanese study also presented similar results, in which it was suggested that high temperatures resulted in increased tear evaporation and led to DES [21]. However, in our study, after adjusting for confounding factors, the effect of temperature on DES was no longer significant (Table 5). Since the distribution of temperature in Taiwan is very similar to that of air pollution concentration, the effect of temperature on dry eye is considered to be less important than that of air pollution in this study. However, another study showed that a threefold increase in tear evaporation rate was observed as the ambient temperature increased from 5 to 25 °C [37]. Further research is needed to study the effect of temperature on DES.

The factors previously identified as conferring an increased risk of DES were immune-mediated diseases such as allergy or arthritis [11,14,38]. From a census of DES in Sydney on residents aged 49 years or older, the prevalence of DES was found to be higher in females than in males; meanwhile, the population with a history of arthritis had a significant 1.8-fold increased risk of DES [38]. Some autoimmune diseases accompanying connective tissue diseases could affect the lacrimal glands, resulting in dry mouth and dry eyes [39]. In addition, the mechanism linking immune-mediated diseases with the prevalence of DES might be through the change of inflammatory mediators on the ocular surface [40]. In a 2020 study, Aluru et al. revealed the change of DES-RA-specific tear protein using 2D-DIGE-based proteomic analysis [41]. Furthermore, anti-inflammatory agents such as corticosteroid used in the treatment of arthritis and allergy may have adverse effects on the eye [38,42]. In this study, we did not acquire the history of medication from the self-reported questionnaires because the study population here consisted of the general public, potentially with a limited knowledge of the detailed names of medications. In addition, in the study by Moss et al., a history of arthritis was found to be an independent factor for DES, and use of aspirin did not interact with a history of arthritis in affecting the prevalence [11].

A few studies have revealed the associations of body mass and blood pressure with DES. For example, in the above-mentioned study by Moss et al. (2000), body mass and blood pressure were found not to be significant risk factors for DES [11]. However, our study showed that, in women with higher SBP, DBP, and BMI, as well as higher waist-to-hip ratio (per unit increment), there were significant protective effects against the risk of DES, with PRs ranging from 0.86 to 0.93. The same result of high BMI being a preventive factor (PR, 0.69; 95% CI: 0.48–1.01) against DES in women was revealed by a Japanese study [12]. A possible explanation for this is that these individuals are more likely to lead a sedentary lifestyle at home, which prevents exposure to outdoor air pollutants resulting in a lower prevalence of DES.

Our study had several strengths. We used the nationwide database of the Taiwan Biobank and collected data from local monitoring stations (2006–2018) with a hybrid kriging/land-use regression (LUR) model to assess the relationship between exposure to air pollution with DES. The sample group was large and the subjects from the Taiwan Biobank were relatively healthy and had better than average quality of life. The data on the levels of exposure to air pollutants were obtained from local monitoring stations with the hybrid kriging/land use regression (LUR) model, which considered the land use conditions, had improved accuracy in predicting the variation of various air pollutants. The model has high explanatory power (R^2^ > 0.85) [24]. Nevertheless, there are several limitations associated with this study. First, although the data from the monitoring stations were those obtained nearest to the individuals’ residences, they did not reflect exposure at the individual level, and indoor pollutants may also have been neglected. Second, despite a meticulous study design with adequate control of confounding factors, it is possible that unmeasured or unknown confounders induced bias in the results. Furthermore, the diagnosis of DES by using a self-reported questionnaire could be a limitation of this study. The prevalence rate of DES in the present study was approximately 11% (Table S1). It is within the range of published survey data in Taiwan (4.87–34%) [8,9]. Comparing with data from other published papers, the reported prevalence rates of DES are in the range of 4.87–61.57% [6,7,9,43]. The prevalence rate of 11% is within this range. The reason why the range of prevalence rates of DES is so large is mainly related to the differences in the age distribution of the subjects, as well as how DES was diagnosed, such as using ICD-9 codes or clinical examination tools.

## 5. Conclusions

The present study specifically addressed the relationship between women’s exposure to air pollutants and the prevalence of DES. The results of this study demonstrated that, apart from the conventional risk factors of dry eye syndrome (hormone therapy, arthritis, and allergy), women should also be aware of the adverse effect of exposure to ambient NO_2_ on DES. Although the annual average NO_2_ exposure concentration was within the range of the Taiwanese national air quality standard (<50 ppb), we still observed an influence of it on DES in this study. Therefore, we recommend that women, especially postmenopausal women, take some personal protective measures against air pollution when going outside.

## Figures and Tables

**Table 1:** Descriptive information of baseline characteristics of study participants.

Variable	Case	Control	PR (95%CI) ^a^
	*n* = 1376	*n* = 5504	
Age, Mean ± SD	53.89 ± 9.95	53.55 ± 9.94	
30–40	148 (10.76)	592 (10.76)	
40–50	288 (20.93)	1152 (20.93)	
50–60	485 (35.25)	1940 (35.25)	
60–70	455 (33.07)	1820 (33.07)	
Education			
Elementary school or below	138 (10.03)	552 (10.03)	
High school	612 (44.48)	2448 (44.48)	
College or above	626 (45.49)	2504 (45.49)	
Marriage			
Single	142 (10.33)	548 (9.97)	1.09 (0.89–1.35)
Married	965 (70.18)	3970 (72.22)	1
Divorce	268 (19.49)	979 (17.81)	1.11 (0.95–1.30)
Resident region			
Northern	654 (47.53)	2822 (51.27)	1.28 (0.93–1.76)
Central	337 (24.49)	1131 (20.55)	1.67 (1.21–2.32) **
Southern	336 (24.42)	1278 (23.22)	1.46 (1.05–2.02) *
East	49 (3.56)	273 (4.96)	1
Job			
No	279 (56.14)	1057 (51.54)	1
Yes	218 (43.86)	994 (48.46)	0.85 (0.69–1.04)
Income			
≤3 thirty thousands	265 (57.73)	1116 (57.70)	1
3–10 thirty thousands	181 (39.43)	755 (39.04)	1.01 (0.82–1.26)
>10 thirty thousands	13 (2.83)	63 (3.26)	0.88 (0.48–1.62)
Menopause			
No	484 (35.17)	2066 (37.55)	1
Yes	892 (64.83)	3436 (62.45)	1.13 (0.92–1.39)
Hormone supplementation			
No	374 (74.06)	1689 (81.55)	1
Yes	131 (25.94)	382 (18.45)	1.55 (1.22–1.95) **
Cigarette smoking			
No	1351 (98.18)	5390 (97.93)	1
Yes	25 (1.82)	114 (2.07)	0.90 (0.58–1.40)
Alcohol drinking			
No	1358 (98.69)	5412 (98.38)	1
Yes	18 (1.31)	89 (1.62)	0.82 (0.49–1.36)
Sport habits			
No	688 (50.00)	2893 (52.60)	1
Yes	688 (50.00)	2607 (47.40)	1.10 (0.97–1.24)
Tea drinking			
No	368 (73.45)	1538 (74.59)	1
Yes	133 (26.55)	524 (25.41)	1.07 (0.86–1.34)
Coffee drinking			
No	320 (63.87)	1280 (62.08)	1
Yes	181 (36.13)	782 (37.92)	0.93 (0.76–1.14)

PR: prevalence ratio; ^a^ Age and education were adjusted in the multiple logistic regression. * 0.01 < *p* < 0.05; ** *p* < 0.01.

**Table 2:** Associations between clinical biochemistry index and individual histories of diseases as well as dry eye syndrome.

Variable	Case	Control	PR (95%CI) ^a^
	*n* = 1376	*n* = 5504	
BMI (kg/m^2^), per SD increment	23.26 ± 3.34	23.66 ± 3.61	0.88 (0.83–0.94) **
Waist to hip ratio, per SD increment	0.85 ± 0.07	0.85 ± 0.07	0.93 (0.88–0.99) *
SBP (mmHg), per SD increment	116.12 ± 17.26	118.03 ± 18.23	0.86 (0.80–0.92) **
DBP (mmHg), per SD increment	69.99 ± 9.99	71.03 ± 10.07	0.89 (0.84–0.95) **
Fasting glucose (mg/dL), per SD increment	94.80 ± 16.78	95.37 ± 19.80	0.96 (0.90–1.03)
HbA1c (%), per SD increment	5.75 ± 0.66	5.77 ± 0.78	0.96 (0.90–1.02)
total Cholesterol (mg/dL), per SD increment	200.88 ± 34.33	200.76 ± 35.85	1.00 (0.94–1.06)
TG (mg/dL), per SD increment	104.43 ± 72.06	105.22 ± 72.44	0.98 (0.93–1.05)
HDL (mg/dL), per SD increment	58.14 ± 13.37	57.73 ± 13.19	1.03 (0.97–1.09)
LDL (mg/dL), per SD increment	122.66 ± 30.81	122.72 ± 31.90	0.99 (0.93–1.05)
TC/HDL ratio, per SD increment	3.60 ± 0.91	3.63 ± 0.94	0.97 (0.91–1.03)
eGFR (ml/min per 1.73 m^2^), per SD increment	102.60 ± 13.81	103.06 ± 13.35	0.98 (0.91–1.06)
FEV/FVC (%), per SD increment	72.00 ± 18.39	71.70 ± 18.74	1.02 (0.94–1.10)
Individual histories of diseases			
Allergy			
No	1184 (86.55)	4985 (89.34)	1
Yes	184 (13.45)	584 (10.66)	1.30 (1.09–1.55) **
Osteoporosis			
No	1229 (89.64)	5084 (92.72)	1
Yes	142 (10.36)	399 (7.28)	1.46 (1.19–1.80) **
Arthritis			
No	1203 (87.75)	5123 (93.43)	1
Yes	168 (12.25)	360 (6.57)	2.00 (1.64–2.43) **
Gout			
No	1358 (99.05)	5435 (99.12)	1
Yes	13 (0.95)	48 (0.88)	1.06 (0.57–1.97)
Asthma			
No	1322 (96.64)	5330 (97.05)	1
Yes	46 (3.36)	162 (2.95)	1.15 (0.82–1.60)

PR: prevalence ratio; Continuous data were presented with Mean ± SD. ^a^ Age and education were adjusted in the multiple logistic regression. * 0.01 < *p* < 0.05; ** *p* < 0.01.

**Table 3:** The effects of various air pollutants on the risk of dry eye syndrome using logistic regression.

Air Pollutants	Case	Control	PR (95%CI) ^a^	PR (95%CI) ^b^
PM_2.5_ (μg/m^3^), per SD increment	31.16 ± 5.67	30.68 ± 5.78	1.09(1.02–1.15) **	1.04(0.94–1.15)
<28.13	446 (32.41)	2017 (36.65)	Reference	Reference
≥28.13	930 (67.59)	3487 (63.35)	1.21 (1.07–1.37) **	1.09 (0.88–1.36)
SO_2_ (ppb), per SD increment	4.50 ± 1.53	4.44 ± 1.49	1.05(0.99–1.11)	1.01(0.91–1.11)
<4.61	929 (67.51)	3911 (71.06)	Reference	Reference
≥4.61	447 (32.49)	1593 (28.94)	1.18 (1.04–1.35) **	1.20 (0.98–1.48)
NO_2_ (ppb), per SD increment	19.41 ± 3.33	19.22 ± 3.42	1.06(0.99–1.12)	1.05(0.93–1.19)
<17.02	390 (28.34)	1828 (33.21)	Reference	Reference
≥17.02	986 (71.66)	3676 (66.79)	1.25 (1.10–1.43) **	1.43 (1.15–1.78) **
O_3_ (ppb), per SD increment	26.58 ± 3.24	26.51 ± 3.29	1.02(0.96–1.09)	1.01(0.89–1.15)
<28.89	954 (69.33)	3968 (72.09)	Reference	Reference
≥28.89	422 (30.67)	1536 (27.91)	1.15 (1.01–1.31) *	0.98 (0.79–1.21)
RH (%), per SD increment	77.93 ± 1.91	78.06 ± 1.89	0.93(0.88–0.99) *	0.96(0.86–1.08)
<78.08	614 (44.62)	2180 (39.61)	Reference	Reference
≥78.08	762 (55.38)	3324 (60.39)	0.81 (0.72–0.92) **	0.90 (0.74–1.09)
Temp (°C), per SD increment	22.70 ± 1.08	22.63 ± 1.08	1.07(1.01–1.13) *	1.05(0.95–1.17)
<21.81	205 (14.90)	1081 (19.64)	Reference	Reference
≥21.81	1171 (85.10)	4423 (80.36)	1.40 (1.19–1.65) **	1.44 (1.09–1.89) **

PR: prevalence ratio; CS: case; CN: controls. ^a^ Age and education were adjusted in the multiple logistic regression. ^b^ Age, education, hormone supplement, arthritis, and allergy were adjusted in the multiple logistic regression. * 0.01 < *p* < 0.05; ** *p* < 0.01.

**Table 4:** Interactions of various air pollutants and clinical diseases on risk of dry eye syndrome using multiple logistic regression.

Pollutants	Allergy	PR (95%CI) ^a^	Interaction *p* Value ^b^	Arthritis	PR (95%CI) ^a^	Interaction *p* Value ^b^	Hormone Supplementation	PR (95%CI) ^a^	Interaction *p* Value ^b^
PM_2.5_ (μg/m^3^)			0.7973			0.6271			0.4021
<28.13	No	Reference ^†,^*		No	Reference ^†,^**		No	Reference ^†,^**	
≥28.13	No	1.11 (0.88–1.39)		No	1.07 (0.85–1.35)		No	1.15 (0.90–1.48)	
<28.13	Yes	1.44 (0.86–2.43)		Yes	1.54 (0.85–2.78)		Yes	1.71 (1.14–2.57) **	
≥28.13	Yes	1.47 (1.00–2.15) ^#^		Yes	1.96 (1.32–2.90) **		Yes	1.60 (1.15–2.22) **	
SO_2_ (ppb)			0.2993			0.7514			0.6360
<4.61	No	Reference ^†,^**		No	Reference ^†^ **		No	Reference ^†,^**	
≥4.61	No	1.15 (0.92–1.44)		No	1.22 (0.98–1.51) ^#^		No	1.17 (0.93–1.48)	
<4.61	Yes	1.19 (0.81–1.76)		Yes	1.82 (1.22–2.71) **		Yes	1.43 (1.06–1.93) ^#^	
≧4.61	Yes	1.88 (1.21–2.93) **		Yes	1.99 (1.22–3.26) **		Yes	1.88 (1.30–2.72) **	
NO_2_ (ppb)			0.3186			0.0698			0.6130
<17.02	No	Reference ^†,^**		No	Reference ^†,^**		No	Reference ^†,^**	
≥17.02	No	1.37 (1.09–1.73) **		No	1.33 (1.05–1.67) **		No	1.39 (1.08–1.78) ^#^	
<17.02	Yes	1.06 (0.60–1.90)		Yes	1.07 (0.57–2.03)		Yes	1.34 (0.86–2.10)	
≥17.02	Yes	2.05 (1.41–2.99) **		Yes	2.80 (1.88–4.16) **		Yes	2.13 (1.54–2.93) **	
O_3_ (ppb)			0.3406			0.5012			0.6984
<28.89	No	Reference		No	Reference ^†,^*		No	Reference ^†,^**	
≥28.89	No	0.94 (0.75–1.18)		No	0.95 (0.76–1.19)		No	1.00 (0.79–1.27)	
<28.89	Yes	1.22 (0.84–1.77)		Yes	1.61 (1.08–2.41) *		Yes	1.53 (1.15–2.04) **	
≥28.89	Yes	1.54 (0.97–2.44) ^#^		Yes	1.91 (1.18–3.09) **		Yes	1.39 (0.95–2.04) ^#^	
RH (%)			0.3648			0.7979			0.5390
<78.08	No	Reference		No	Reference ^†,#^		No	Reference ^†,^*	
≥78.08	No	0.93 (0.75–1.15)		No	0.91 (0.73–1.12)		No	0.87 (0.69–1.09)	
<78.08	Yes	1.57 (1.03–2.38) *		Yes	1.80 (1.17–2.78) **		Yes	1.37 (0.97–1.94) ^#^	
≥78.08	Yes	1.11 (0.73–1.69)		Yes	1.50 (0.95–2.38) ^#^		Yes	1.38 (1.00–1.90) ^#^	
Temperature (°C)			0.3582			0.1673			0.8472
<21.81	No	Reference ^†,^**		No	Reference ^†,^**		No	Reference ^†,^**	
≥21.81	No	1.37 (1.03–1.83) *		No	1.34 (1.00–1.78) *		No	1.46 (1.06–2.01) *	
<21.81	Yes	0.93 (0.40–2.19)		Yes	1.01 (0.43–2.38)		Yes	1.57 (0.89–2.75)	
≥21.81	Yes	1.96 (1.32–2.90) **		Yes	2.58 (1.70–3.90) **		Yes	2.15 (1.48–3.12) **	

PR: prevalence ratio; ^a^ Age, education, hormone supplement, arthritis and allergy were adjusted in the multiple logistic regression. ^b^ Interaction *p* values were calculated through multiplicative models. ^†^ trend *p* values with significance were shown. ^#^ 0.05 < *p* < 0.1; * 0.01 < *p* < 0.05; ** *p* < 0.01.

**Table 5:** Stepwise logistic regression analysis.

Variable	PR (95%CI)	*p* Value
Hormone supplementation (Yes vs. No)	1.47 (1.17–1.86)	0.0012
DBP (continuous) (mmHg)	0.99 (0.98–1.00)	0.0042
Allergy (Yes vs. No)	1.36 (1.01–1.82)	0.0422
Arthritis (Yes vs. No)	1.76 (1.29–2.41)	0.0004
NO_2_ (ppb) ( >= 17.02 vs. < 17.02)	1.43 (1.15–1.78)	0.0015

PR: prevalence ratio. All relevant factors in Table 1, Table 2 and Table 3 were included in the stepwise logistic regression model.

## Data Availability

The data were obtained from the Taiwan Biobank, but restrictions apply to the availability of these data, which were used under license for the current study, and so are not publicly available. Any detail for data requests can be through the Taiwan Biobank (https://www.twbiobank.org.tw/new_web/about.php, accessed on 17 May 2019.

## Supplementary Materials

The following are available online at https://www.mdpi.com/article/10.3390/ijerph18136860/s1. Table S1: Descriptive information of baseline characteristics of study participants. Table S2: Sensitivity analysis for the effects of various air pollutants on the risk of dry eye syndrome 1 year, 3 years, or 5 years exposure duration of air pollutants before the survey day.
