# Supplementary material for: Exposure to Ambient NO2 Increases the Risk of Dry Eye Syndrome in Females: An 11-Year Population-Based Study
Source: Int J Environ Res Public Health. 2021 Jun 26;18(13):6860. doi: 10.3390/ijerph18136860 (PMC8296916; doi:10.3390/ijerph18136860)
Supplement: Supplementary file 1 [file ijerph-18-06860-s001.zip › ijerph-1240672-supplementary.pdf]

Supplement Table 1 Descriptive information of baseline characteristics of study participants

| Variable | case | control | p values [a] | PR(95%CI) [b] |
|---|---|---|---|---|
| | n=2096 | n=17210 | | |
| Age, Mean± SD | 54.79±10.06 | 49.90±10.80 | | |
| 30-40 | 214 (10.21) | 3719 (21.61) | <0.0001 | 1.00 |
| 40-50 | 387 (18.46) | 4631 (26.91) | | 1.45 (1.22-1.72) ** |
| 50-60 | 673 (32.11) | 4877 (28.34) | | 2.44 (2.07-2.86) ** |
| 60-70 | 822 (39.22) | 3983 (23.14) | | 3.68 (3.14-4.32) ** |
| Sex | | | | |
| Male | 570 (27.19) | 8971 (52.13) | <0.0001 | 1.00 |
| Female | 1526 (72.81) | 8239 (47.87) | | 2.98 (2.69-3.30) ** |
| Education | | | | |
| Elementary school or below | 175 (8.35) | 917 (5.33) | <0.0001 | 1.00 |
| High school | 862 (41.15) | 6609 (38.42) | | 1.11 (0.92-1.33) |
| College or above | 1058 (50.50) | 9677 (56.25) | | 1.30 (1.08-1.57) ** |
| Resident region | | | | |
| Northern | 969 (46.23) | 8415 (48.90) | 0.0011 | 1.00 |
| Central | 525 (25.05) | 3756 (21.82) | | 1.33 (1.18-1.49) ** |
| Southern | 529 (25.24) | 4265 (24.78) | | 1.13 (1.01-1.27) * |
| East | 73 (3.48) | 774 (4.50) | | 0.82 (0.64-1.06) |

PR: prevalence ratio.

[a] p values calculated by Chi-squares tests.

[b] Age and sex were adjusted in the multiple logistic regression.

* 0.01<p<0.05; ** p<0.01.

Supplement Table 2 Sensitivity analysis for the effects of various air pollutants on the risk of dry eye syndrome 1 year, 3 years, or 5 years exposure duration of air pollutants before the survey day

| Air pollutants | Case | Control | PR(95%CI)[a] | PR(95%CI)[b] |
|---|---|---|---|---|
| One year before the survey day | | | | |
| $PM_{2.5}$ (μg/ $m^3$), per SD increment | 30.27±5.05 | 29.77±5.16 | 1.10(1.04-1.17) ** | 1.06(0.95-1.17) |
| <27.38 | 402 (29.22) | 1876 (34.08) | Reference | Reference |
| ≧27.38 | 974 (70.78) | 3628 (65.92) | 1.25 (1.10-1.42) ** | 1.15 (0.93-1.41) |
| $SO_2$ (ppb), per SD increment | 3.65±0.92 | 3.60±0.90 | 1.05(0.99-1.12) # | 1.02(0.93-1.12) |
| <3.80 | 958 (69.62) | 3988 (72.46) | Reference | Reference |
| ≧3.80 | 418 (30.38) | 1516 (27.547) | 1.15 (1.01-1.31) * | 1.18 (0.96-1.45) |
| $NO_2$ (ppb), per SD increment | 15.24±2.88 | 15.10±2.91 | 1.05(0.99-1.11) | 1.06(0.95-1.18) |
| <14.66 | 569 (41.35) | 2459 (44.68) | Reference | Reference |
| ≧14.66 | 807 (58.65) | 3045 (55.32) | 1.14 (1.01-1.29) * | 1.29 (1.06-1.59) * |
| $O_3$ (ppb), per SD increment | 28.33±3.53 | 28.20±3.59 | 1.04(0.98-1.11) | 1.02(0.89-1.16) |
| <30.18 | 799 (58.07) | 3415 (62.05) | Reference | Reference |
| ≧30.18 | 577 (41.93) | 2089 (37.95) | 1.19 (1.06-1.34) ** | 1.06 (0.87-1.29) |
| Three years before the survey day | | | | |
| $PM_{2.5}$ (μg/ $m^3$), per SD increment | 30.5±5.19 | 30.05±5.32 | 1.09(1.03-1.15) ** | 1.03(0.93-1.15) |
| <26.98 | 366 (26.60) | 1658 (30.12) | Reference | Reference |
| ≧26.98 | 1010 (73.40) | 3846 (69.88) | 1.19 (1.04-1.36) * | 1.03 (0.83-1.28) |
| $SO_2$ (ppb), per SD increment | 3.8±1.09 | 3.75±1.06 | 1.05(0.99-1.11) | 1.02(0.93-1.12) |
| <4.07 | 957 (69.55) | 3984 (72.38) | Reference | Reference |
| ≧4.07 | 419 (30.45) | 1520 (27.62) | 1.15 (1.01-1.31) * | 1.17 (0.96-1.44) |
| $NO_2$ (ppb), per SD increment | 16.39±3.05 | 16.21±3.1 | 1.06(1.00-1.12) # | 1.06(0.95-1.18) |
| <14.81 | 416 (30.23) | 1942 (35.28) | Reference | Reference |
| ≧14.81 | 960 (69.77) | 3562 (64.72) | 1.25 (1.10-1.43) ** | 1.44 (1.15-1.79) ** |
| $O_3$ (ppb), per SD increment | 27.92±3.23 | 27.82±3.27 | 1.04(0.98-1.10) | 1.01(0.89-1.14) |
| <29.76 | 804 (58.43) | 3436 (62.43) | Reference | Reference |
| ≧29.76 | 572 (41.57) | 2068 (37.57) | 1.19 (1.06-1.35) ** | 1.06 (0.87-1.29) |
| Five years before the survey day | | | | |
| $PM_{2.5}$ (μg/ $m^3$), per SD increment | 30.60±5.38 | 30.13±5.50 | 1.09(1.03-1.16) ** | 1.04(0.94-1.15) |
| <25.19 | 162 (11.77) | 834 (15.15) | Reference | Reference |
| ≧25.1 | 1214 (88.23) | 4670 (84.85) | 1.33 (1.11-1.60) ** | 1.08 (0.79-1.49) |
| $SO_2$ (ppb), per SD increment | 4.01±1.21 | 3.95±1.18 | 1.05(0.99-1.11) # | 1.02(0.93-1.12) |
| <4.14 | 934 (67.88) | 3907 (70.98) | Reference | Reference |
| ≧4.14 | 442 (32.12) | 1597 (29.02) | 1.16 (1.02-1.32) * | 1.19 (0.97-1.45) |
| $NO_2$ (ppb), per SD increment | 17.08±3.15 | 16.9±3.21 | 1.06(0.99-1.12) # | 1.06(0.94-1.19) |

| | | | | |
|---|---|---|---|---|
| <15.37 | 461 (33.50) | 2087 (37.92) | Reference | Reference |
| ≧15.37 | 915 (66.50) | 3417 (62.08) | 1.21 (1.07-1.37) ** | 1.37 (1.11-1.68) ** |
| $O_3$ (ppb), per SD increment | 27.69±3.25 | 27.6±3.3 | 1.03(0.97-1.10) | 1.01(0.89-1.15) |
| <30.30 | 970 (70.49) | 4071 (73.96) | Reference | Reference |
| ≧30.30 | 406 (29.51) | 1433 (26.04) | 1.20 (1.05-1.37) ** | 0.97 (0.78-1.21) |

PR: prevalence ratio; CS: case; CN: controls. [a] Age and education were adjusted in the multiple logistic regression. [b] Age, education, hormone supplement, arthritis and allergy were adjusted in the multiple logistic regression. # 0.05<p<0.1; * 0.01<p<0.05; ** p<0.01
